# Comprehensive analysis of necroptosis-related genes in renal ischemia-reperfusion injury

**DOI:** 10.3389/fimmu.2023.1279603

**Published:** 2023-10-27

**Authors:** Shuai Li, Weixun Zhang, Xiaopeng Hu

**Affiliations:** ^1^ Department of Urology, Beijing Chao-Yang Hospital, Capital Medical University, Beijing, China; ^2^ Institute of Urology, Capital Medical University, Beijing, China

**Keywords:** kidney transplantation, necroptosis, inflammatory cells, oxidative stress, ischemia-reperfusion injury, delayed graft function, predictive model

## Abstract

**Background:**

Oxidative stress is the primary cause of ischemia-reperfusion injury (IRI) in kidney transplantation, leading to delayed graft function (DGF) and implications on patient health. Necroptosis is believed to play a role in renal IRI. This research presents a comprehensive analysis of necroptosis-related genes and their functional implications in the context of IRI in renal transplantation.

**Methods:**

The necroptosis-related differentially expressed genes (NR-DEGs) were identified using gene expression data from pre- and post-reperfusion renal biopsies, and consensus clustering analysis was performed to distinguish necroptosis-related clusters. A predictive model for DGF was developed based on the NR-DEGs and patients were divided into high- and low-risk groups. We investigated the differences in functional enrichment and immune infiltration between different clusters and risk groups and further validated them in single-cell RNA-sequencing (scRNA-seq) data. Finally, we verified the expression changes of NR-DEGs in an IRI mouse model.

**Results:**

Five NR-DEGs were identified and were involved in various biological processes. The renal samples were further stratified into two necroptosis-related clusters (C1 and C2) showing different occurrences of DGF. The predictive model had a reliable performance in identifying patients at higher risk of DGF with the area under the curve as 0.798. Additionally, immune infiltration analysis indicated more abundant proinflammatory cells in the high-risk group, which was also found in C2 cluster with more DGF patients. Validation of NR-DEG in scRNA-seq data further supported their involvement in immune cells. Lastly, the mouse model validated the up-regulation of NR-DEGs after IR and indicated the correlations with kidney function markers.

**Conclusions:**

Our research provides valuable insights into the identification and functional characterization of NR-DEGs in the context of renal transplantation and sheds light on their involvement in immune responses and the progression of IRI and DGF.

## 1 Introduction

Kidney transplantation (KT) is the optimal renal replacement treatment for end-stage renal disease, associated with lower mortality and enhanced quality of life in comparison to chronic dialysis treatment (1). Recently, the survival rates for patients and grafts have exceeded 96% and 91% correspondingly in their first year (2). However, the incidence of renal insufficiency and comorbidities after KT remains high, leading to potential loss of transplanted kidney function. Delayed graft function (DGF) is among the most frequent postoperative complications, with prevalence varying between 2% to 50% in different types of kidney grafts, which may be related to the use of expanded criteria donors and deceased cardiac dead (DCD) donors (3). At present, the standardized definition of DGF is controversial and cannot provide early warning signs (4, 5). Therefore, there is still a clinical demand for a non-invasive, robust, and more reliable diagnostics method to detect DGF.

Ischemia-reperfusion injury (IRI) induced by oxidative stress, is the primary cause of DGF, acute rejection, and chronic rejection (6), and is inevitable during KT (7). The imbalance between oxygen supply and demand could cause oxidative metabolic disorders, resulting in the death of tubular epithelial cells (TECs) and impairment of kidney function (8). During both the ischemic phase and subsequent reperfusion, cellular damage takes place and causes the loss of cellular polarity, impaired brush border, decreased intercellular adhesion, and cell death (3, 8). Necrosis, traditionally considered as a non-programmed cell death, is the main form of tubular cell death of renal IRI and acute kidney injury (AKI) (8–10). However, emerging evidence suggests the existence of highly regulated forms of non-apoptotic cell death with necrotic characteristics, known as regulated necrosis (RN) (6). RN can take various forms, including necroptosis, ferroptosis, necrosis driven by mitochondrial permeability transition (MPT), pyroptosis, and parthanatos. Among these, necroptosis and ferroptosis are the most extensively studied forms in the context of renal IRI. Necroptosis is characterized by its dependency on the kinase domain of receptor-interacting protein kinase (RIPK)-3 and the phosphorylation of mixed lineage kinase domain-like protein (MLKL) (9). It has been reported to contribute to several models of renal injury, including IRI (6), cisplatin-induced AKI (11), and contrast-induced nephropathy (12). Although numerous studies have explored potential protective therapies for renal IRI (13), their practical value remains controversial, and none of them has been translated to clinical application. Given the development of massive sequencing and genetic diagnosis techniques, it is essential to investigate the role of necroptosis in gene-based diagnostic and therapeutic strategies for DGF. It has been reported that necroptosis-related genes (NRGs) are upregulated early in the renal allograft and serve as risk factors for subsequent DGF (14). However, the association between NRGs and specific cell types or their role in the induction of specific immune responses during the process of renal IRI remains ambiguous.

In this study, we aimed to identify potential target genes by intersecting necroptosis-related genes and differentially expressed genes (DEGs) obtained from two gene expression omnibus (GEO) databases of KT. Based on the expression levels of necroptosis-related DEGs (NR-DEGs), we stratified the samples into two clusters with distinct molecular and clinical features. Subsequently, we developed a diagnostic model for predicting the occurrence of DGF based on the targeted NR-DEGs and categorized the samples into high- and low-risk groups. Furthermore, we conducted functional enrichment and immune-infiltration analyses to explore the potential mechanisms involved. Single-cell RNA-sequencing (scRNA-seq) data and cell-cell communication analyses were further utilized to investigate the relationship between necroptosis, NR-DEGs, and immune cells. Finally, we validated our findings using a mice kidney IRI model. In brief, our study provides novel necroptosis-related biomarkers for the early diagnosis of DGF after KT and enables the discrimination of patients at different risk levels.

## 2 Materials and methods

### 2.1 The gathering and analysis of bulk RNA-Seq data

The RNA-seq datasets utilized in this study were acquired from the GEO database (https://www.ncbi.nlm.nih.gov/geo/). Specifically, we accessed the GSE43974 dataset, comprising 188 renal biopsies before retrieval and 203 biopsies after reperfusion obtained from brain-dead (BD) kidney donors, DCD donors, and living donors. This dataset was used for the identification of hub genes and the development of the predictive model. Additionally, we utilized the GSE126805 dataset, which included protocol biopsies collected at different time points from 42 kidney transplant recipients, for validation purposes. The microarray datasets based on Illumina platforms were subjected to log2 transformation and normalized by R package “limma”. The demographic characteristics are detailed in **Table S1**.

### 2.2 Identification of the necroptosis-related genes

From the GeneCards database (https://auth.lifemapsc.com/), a collection of 114 genes associated with necroptosis was acquired with a relevance score > 1 (**Table S2**). IRI-related DEGs between pre- and post-reperfusion from three different types of donor types (BD, DCD, and living donors) in the GSE43974 dataset were screened by “limma” R package with adj. p < 0.05 and |logFC| ≥ 0.5, respectively. To screen out the NR-DEGs, a Venn analysis was undertaken to identify the intersected genes of the above three different analyses and necroptosis-related genes.

### 2.3 Analysis of functional enrichment and immune infiltration

The Gene Ontology (GO) and Kyoto Encyclopedia of Genes and Genomes (KEGG) enrichment analyses of the common IRI-related DEGs among three donor types were performed by “clusterProfiler” R package (15–17). The evaluation of the abundance of 22 different immune cells was conducted using CIBERSORT (http://cibersort.stanford.edu/) (18).

### 2.4 Necroptosis-based consensus clustering analysis

To determine the molecular patterns associated with necroptosis in samples of IRI, consensus clustering analysis was performed based on the above NR-DEGs using the “ConsensusClusterPlus” R package (19). To ensure classification stability, 80% item resampling and a maximum evaluated k of 9 were used. The principal component analysis (PCA) was performed to validate cluster results.

### 2.5 Establishment of the predictive model

The machine learning algorithm was performed in the GSE43974 dataset to screen out necroptosis-related hub genes and construct a predictive model. The least absolute shrinkage and selection operator (LASSO) regression analysis, a variable selection method for regression models, was utilized to eliminate less informative features (20). The regression coefficients of each gene were estimated by the least squares method based on parameters obtained from the cross-validation. We conducted the LASSO regression analysis with 10-fold cross-validation (utilizing “glmnet” R package). The area under the receiver operator characteristic curve (AUROC) of the predictive model was evaluated through the “ROCR” R package.

Based on the necroptosis-related score derived from the risk prediction model, the samples of IRI were stratified into high- and low-risk groups, respectively.

### 2.6 Obtaining and analyzing scRNA-seq data

The scRNA-seq dataset GSE171639 consisting of two mice kidney samples following ischemic reperfusion or sham surgery was obtained from the GEO database (21). The kidneys were subjected to bilateral clamping of the renal pedicle for a duration of 30 minutes, after which reperfusion was allowed for 6-7 hours. For further verification, additional scRNA-seq data of samples from mice kidneys after 27min ischemia or without any surgery were used (GSE193649) (22). The additional details of these datasets can be found in **Table S1**.

To preprocess the data, we utilized the “Seurat” R package. Several quality control measures were applied, including calculating the percentage of gene numbers, cell counts, and mitochondria sequencing counts. Cells exceeding a mitochondrial content of 50%, having fewer than 200 genes, and falling within the lower 10% and top 5% percentiles of unique gene distribution were removed for GSE171639 dataset (**Figures S1A, B**). Afterward, the cells were normalized for sequencing depth by utilizing the “NormalizeData” function with the default method of “LogNormalize”. Using the “FindVariableFeatures” function, we detected the top 2,000 highly variable genes and then scaled them through the “ScaleData” function. Next, PCA was performed to identify significant principal components, and “harmony” R package was employed to integrate the data from each biological individual. Subsequently, the cells were clustered using the “FindNeighbors” and “FindClusters” functions (with a resolution of 0.65 for GSE171639 dataset) and visualized with uniform manifold approximation and projection (UMAP). To identify marker genes for each main cell cluster, the “FindAllMarker” function (|logFC| > 0.3, Minpct = 0.25) was employed. Subsequently, the prominent cell categories were identified according to the markers acquired from the CellMarker2.0 database (23) and previous studies (24–26). The top 3 markers for each cell type were selected and plotted on a heatmap.

### 2.7 Cell-cell communication analysis

The cell-cell communication networks were quantitatively inferred and visualized based on the CellChatDB of ligand-receptor pairs in humans and mice using the scRNA-seq data (GSE171639) and the “CellChat” R package (27). We compared the intercellular communication before and after ischemia-reperfusion (IR), and the minimum threshold of cells required in different cells was set to 10.

### 2.8 Analysis of gene set variation and enrichment

The gene set enrichment analysis for scRNA-seq was performed through GSVA implemented in the “GSVA” package (28). Gene sets were exported using the “GSEABase” R package. The enrichment score of each pathway in the significant cell types was calculated using t-values, and the differences between the sham and IRI groups were compared using the “limma” package. In addition, we visualized the distribution of necroptosis-related pathways with the UMAP function.

### 2.9 Mice and renal IRI model

C57BL/6N mice (8-10 weeks old, male) were obtained from Weitonglihua (Beijing, China) and were kept in a controlled environment free from pathogens. The animal experiment was reviewed and approved by the Ethics Committee of Beijing Chao-Yang Hospital (2021-54). Before the operation, a minimum of one week was given for the mice to adapt to these conditions. Mice underwent IRI (n = 8) as described previously (29). For the procedure, the mice were anesthetized with pentobarbital (60 mg/kg) through an intraperitoneal injection and placed on a thermoregulated heating pad to maintain body temperature at 34 to 36 °C. The right kidney was removed and used as self-control, while the left renal pedicle was clamped for 30 minutes to induce renal ischemia. Subsequently, the clamp was released, allowing tissue reperfusion. Mice were euthanized 24 hours after renal IRI, and both serum and kidney tissues were collected.

### 2.10 Evaluation of kidney damage and renal function

The assessment of kidney injury was conducted based on the levels of blood urea nitrogen (BUN) and serum creatinine (SCr). Serum samples were isolated from the clotted whole blood samples through centrifugation at a speed of 3,000 revolutions per minute for 10 minutes and further subjected to BUN and SCr testing by an automated chemistry analyzer (Chemray 800).

### 2.11 Quantitative real-time PCR

As per the manufacturer’s instructions, total RNA was extracted from the kidney samples using the FastPure Cell/Tissue Total RNA Isolation Kit V2 (Vazyme, RC112-01). The extracted RNA was then reverse transcribed to cDNA by HiScript III RT SuperMix for qPCR kit (Vazyme, R323-01). Subsequently, quantitative PCR (qPCR) was performed on an Applied Biosystems 7500 Fast Real-Time PCR System using the HiScript Q RT SuperMix for qPCR (Vazyme, R122-01). Gapdh was used as an internal reference gene for normalization during qPCR analysis. The primer sequences of the hub genes in this study can be found in **Table S3**.

### 2.12 Statistical analysis

The statistical analysis for this study was conducted using R software (version 4.1.3). To compare the variations in immune cell infiltration and gene expression levels among different groups, either a Student’s t-test or Mann-Whitney U test was employed. A two-sided p value less than 0.05 was considered statistically significant.

## 3 Results

### 3.1 Identification of NR-DEGs and functional enrichment analysis

**Figure 1:**
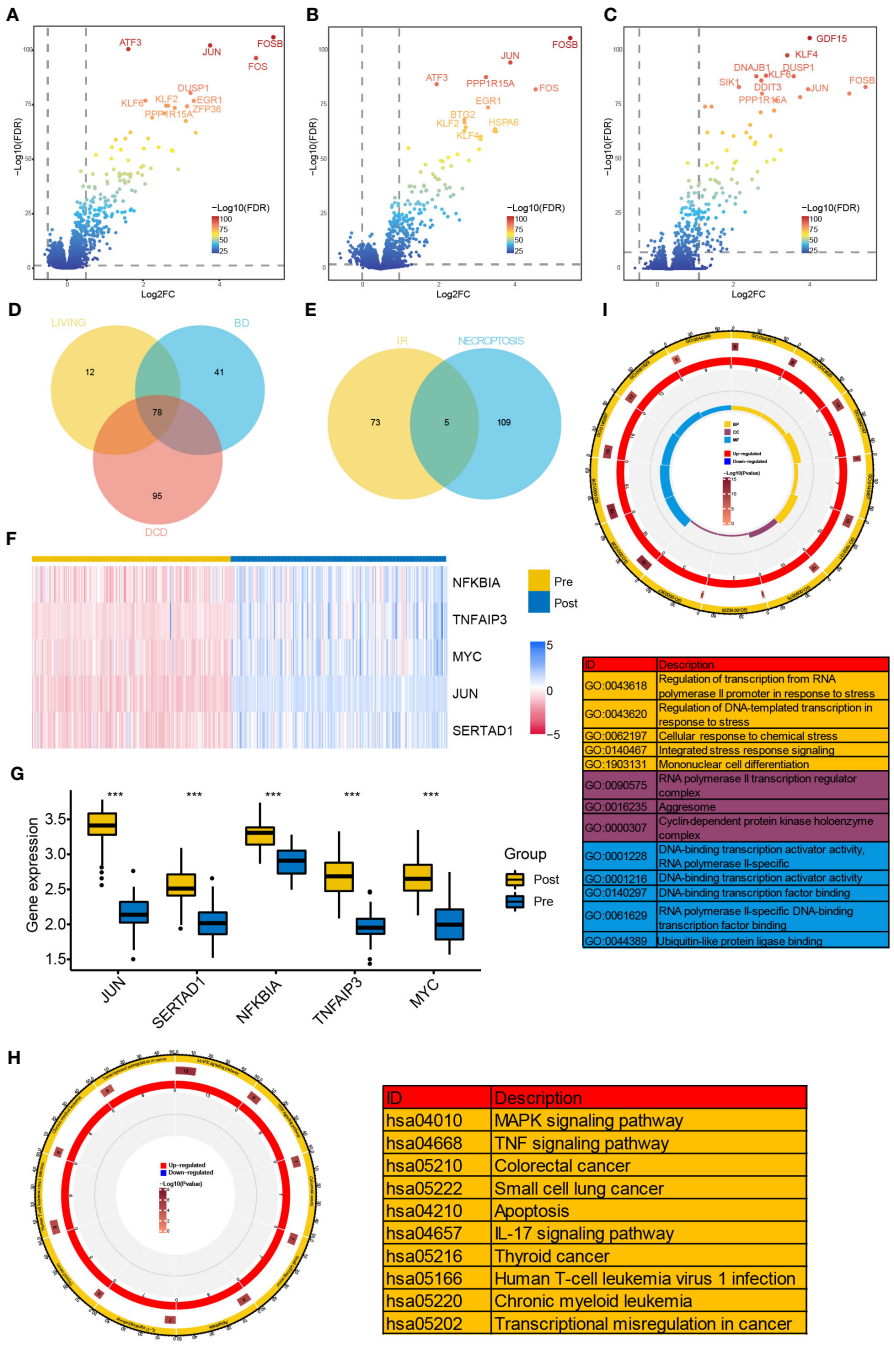
Identification of NR-DEGs and functional enrichment analysis of IRI-related DEGs. **(A–C)** Volcano plots of DEGs between samples before and after reperfusion in brain death **(A)**, cardiac death **(B)**, and healthy living donors **(C)**, respectively. **(D)** The Venn diagram showing the intersection of IRI-related DEGs among three kinds of donors. **(E)** The Veen diagram showing the intersection of DEGs from IR samples and necroptosis-related genes. **(F)** Heatmap of the expression of the 5 NR-DEGs in pre- and post-reperfusion samples in GSE43974. **(G)** Box plots showing the expression levels of the 5 NR-DEGs among pre- and post-reperfusion samples in GSE126805. **(H, I)** Chord plots presenting the distribution of the common IRI-related DEGs in KEGG and GO pathway analysis. NR-DEGs, necroptosis-related differentially expression genes; IRI, ischemia-reperfusion injury; BD, brain death; DCD, deceased cardiac dead; BP, biological process; CC, cellular component; MF, molecular function. *** P value < 0.001.

Differential expression analyses were performed in GSE43974 dataset. As a result, we identified 119, 173, and 90 DEGs from the BD, DCD, and living donor samples, respectively (**Figures 1A–C**). Among these, 78 DEGs were significantly differentially expressed in all three types of donors (**Figure 1D**). To gain insights into the biological function of these common IRI-related DEGs, we conducted functional enrichment analysis based on the GO and KEGG databases. GO analysis encompassed biological process (BP), cellular component (CC), and molecular function (MF). The top five enriched terms of each category are depicted in **Figure 1I**, Notably, the representative enriched terms including regulation of transcription from RNA polymerase II promoter in response to stress (GO: 0043618), RNA polymerase II transcription regulator complex (GO: 0090575), and DNA-binding transcription activator activity (GO: 0001216). Additionally, the KEGG pathway analysis (**Figure 1H**) revealed significant involvement of the IRI-related DEGs in MAPK signaling pathway (hsa: 04010), TNF signaling pathway (hsa: 04668), and IL-17 signaling pathway (hsa: 04657).

Next, we investigated the intersection of the 78 IRI-related DEGs from three types with the 114 NRGs and revealed 5 NR-DEGs (*NFKBIA, TNFAIP3, MYC, JUN, SERTAD1*, **Figure 1E**). We further visualized the expression of the NR-DEGs through a heatmap (**Figure 1F**) and validated these findings in an independent transplantation cohort (GSE126805) as well (**Figure 1G**), indicating that all the identified hub genes were significantly upregulated after IR.

### 3.2 Stratification of IRI samples based on NR-DEGs

**Figure 2:**
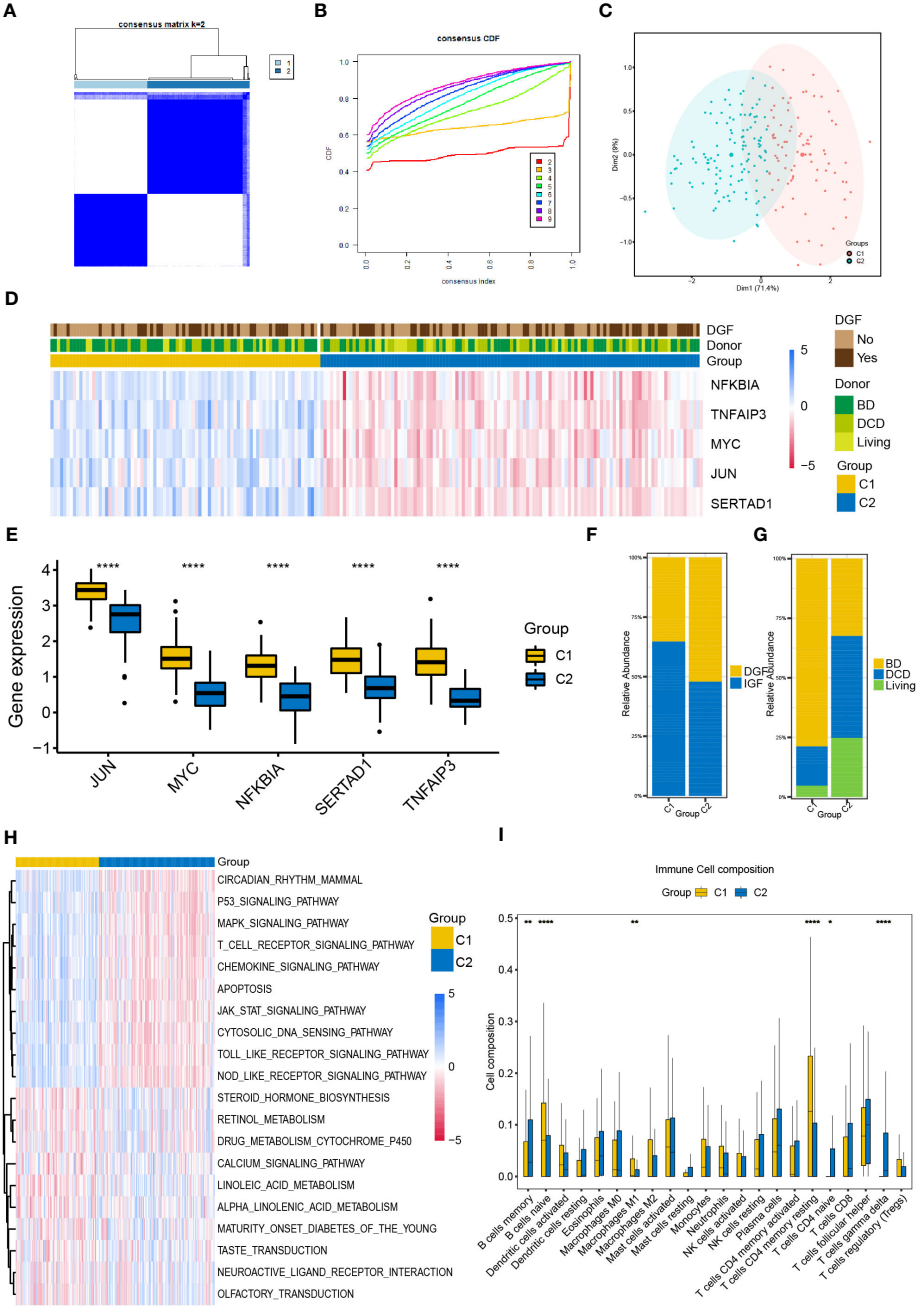
Classification of IR samples into two clusters by consensus clustering analysis. **(A)** Heatmap of consensus clustering analysis based on NR-DEGs. **(B)** Cumulative distribution function curves with k = 2-9. **(C)** PCA analysis of NR-DEGs between two clusters. **(D)** Heatmap showing the association between the expression levels of NR-DEGs and the clinical characteristics of two clusters. **(E)** Box plots showing the expression levels of the 5 NR-DEGs among two clusters in GSE43974. **(F, G)** Histograms comparing the occurrence of DGF and donor types between two clusters. **(H, I)** The GSVA enrichment analysis **(H)** and immune cell infiltration analysis **(I)** among the two clusters. IR, ischemia-reperfusion; NR-DEGs, necroptosis-related differentially expression genes; PCA, principal component analysis; DGF, delayed graft function; GSVA, gene set variation analysis; BD, brain death; DCD, deceased cardiac dead. *P value < 0.05; **P value < 0.01; ****P value < 0.0001.

We performed unsupervised consistent clustering and stratify the renal samples after IR based on the expression levels of the five NR-DEGs. The cumulative distribution curve appeared most horizontal in the middle section at k = 2, and the heatmap of clustering suggested a clear distinction between cluster 1 (C1) and cluster 2 (C2, **Figures 2A, B**). PCA further demonstrated a significant separation of the expression levels of the NR-DEGs between the two clusters (**Figure 2C**). Specifically, the five hub genes expressed at higher levels in C1 group, as evident from the heatmap (**Figure 2D**) and boxplot (**Figure 2E**). As for clinical characteristics, the C1 group had a lower proportion of patients who experienced DGF (35.2% compared to 52.1% in C2, **Figure 2F**). Moreover, the C1 group comprised a significantly lower number of kidney transplant recipients from DCD donors (16.4% compared to 42.7% in C2, **Figure 2G**).

### 3.3 Analysis of immune infiltration of necroptosis-related clusters

To explore the functional differences among the two necroptosis-related clusters, we conducted the GSVA utilizing hallmarks gene set (c2.cp.kegg.v2022.1.Hs.symbols.gmt) and visualized the results using a heatmap (**Figure 2H**). C1 group was positively associated with several pathways, including P53 signaling, MAPK signaling, T cell receptor signaling, chemokine signaling pathways, and apoptosis pathway. In contrast, C2 group showed enrichment in metabolic-related pathways, including steroid hormone biosynthesis, cytochrome P450-mediated drug metabolism, and the pathway of retinol and linoleic acid metabolism.

Furthermore, we investigated the differential relative proportion of immune infiltrating cells between the two clusters. The results revealed that the C1 group exhibited higher abundance of naïve B cells, M1 macrophages, and memory resting CD4 T cells. In contrast, the C2 group had higher infiltration of memory B cells, gamma delta T cells, and naïve CD4 T cells (**Figure 2I**).

### 3.4 Construction and validation of the DGF predictive model

**Figure 3:**
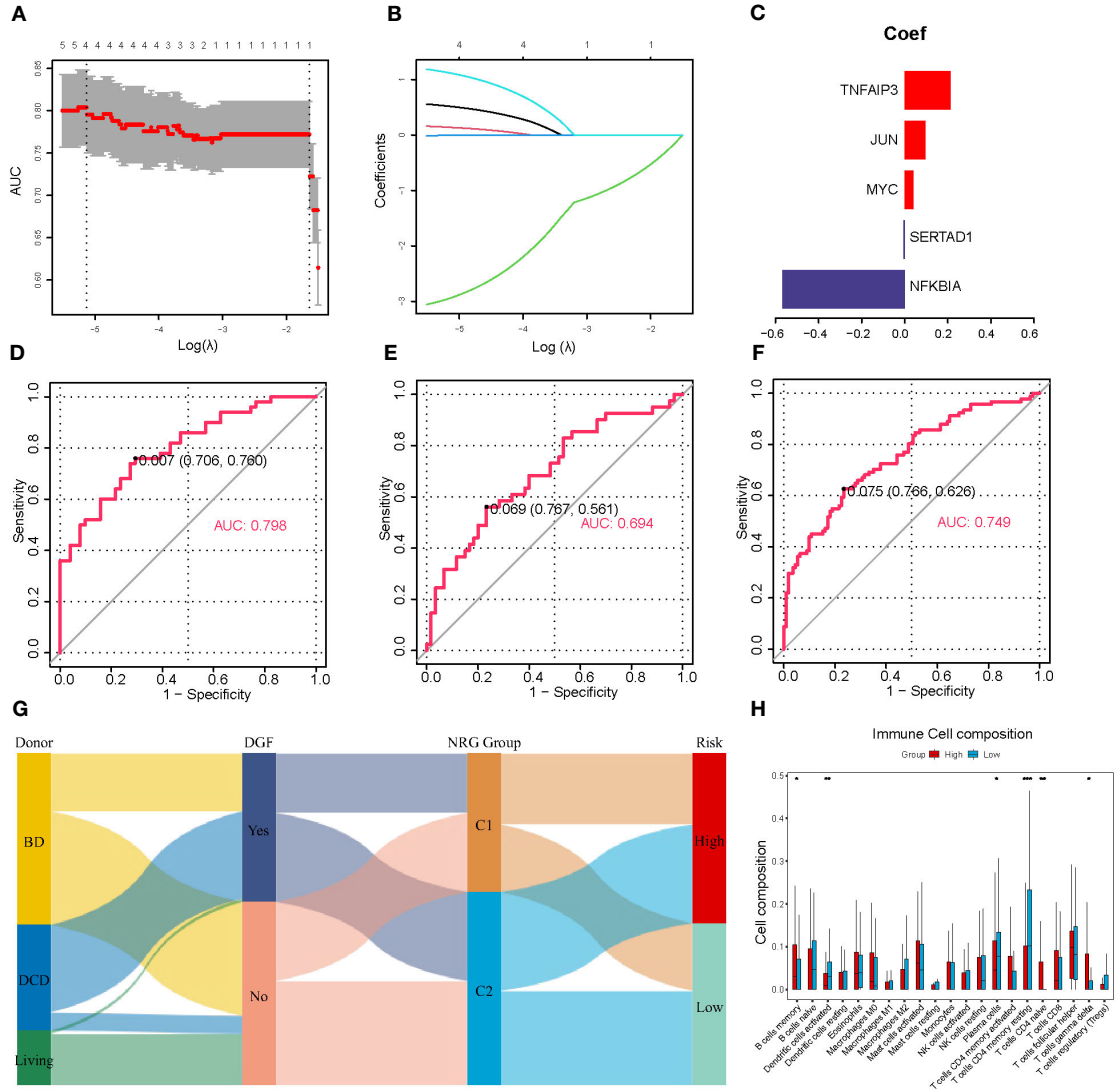
Establishment and validation of the prediction model for DGF. **(A)** Cross-validation plot according to the log of lambda in LASSO regression. **(B)** The coefficients of each NR-DEG over different values of the penalty parameter. **(C)** The final coefficients in the optimal predictive model. **(D-F)** The AUC of the prediction model based on LASSO regression in the discovery **(D)**, validation **(E)**, and whole cohorts **(F)**, respectively. **(G)** Alluvial diagram of donor types, the occurrence of DGF, NRG clusters, and risk groups. **(H)** The immune cell infiltration analysis between two risk groups divided with the risk scores. DGF, delayed graft function; NR-DEGs, necroptosis-related differentially expression genes; AUC, area under the receiver operator characteristic curve; NRG, necroptosis-related gene. *P value < 0.05; **P value < 0.01; ***P value < 0.001.

DGF is a common manifestation seen after renal transplantation and always occurs after severe IRI (30). Therefore, we aimed to develop a predictive model for DGF based on the NR-DEGs from the GSE43974 dataset. We randomly divided these IRI samples into a discovery cohort and an internal validation cohort with a ratio of 7: 3. In the discovery cohort, we used 10-fold cross-validation for LASSO regression to minimize the prediction error and determine the contribution of each gene in the optimal model. The optimal value of log lambda (λ) was indicated by the left dashed vertical line in **Figure 3A**. In this case, all five NR-DEGs (*TNFAIP3, JUN, MYC, SERTAD1*, and *NFKBIA*) were included in the final predictive model (**Figures 3B, C**). The area under the curve (AUC) of the prediction model in the discovery, internal validation, and whole cohorts were 0.798, 0.694, and 0.749 respectively, suggesting that the model was reliable in assessing the risk of DGF after renal transplantation (**Figures 3D–F**).

To further validate the performance of the prediction model, we categorized IRI samples into high- and low-risk groups based on the median risk score derived from the model. **Figure 3H** illustrates the results of immune infiltration analysis. The high-risk group exhibited a higher abundance of immune-related cells, including memory B cells, naïve CD4 T cells, and gamma delta T cells. Conversely, the low-risk group samples tended to have more activated dendritic cells, plasma cells, and memory resting CD4 T cells. These findings were consistent with the comparison between the necroptosis-related clusters, suggesting that the high-risk group samples had a greater accumulation of proinflammatory cell phenotypes. In addition, we utilized a Sankey diagram (**Figure 3G**) to visualize the relationships among various characteristics, including the type of donors, the occurrence of DGF, the necroptosis-related cluster, and the DGF risk.

### 3.5 Validation of the expression of NR-DEGs in scRNA-seq data

As for scRNA-seq data analysis, a total of 19,274 genes and 12,965 cells were included from the GSE171639 dataset after quality filtering and batch effects removal. Among these, 6,697 cells (51.7%) originated from IRI group and 6,268 cells (48.3%) were derived from sham surgery group. By applying PCA and UMAP algorithms, we divided the 12,965 cells into 23 clusters.

**Figure 4:**
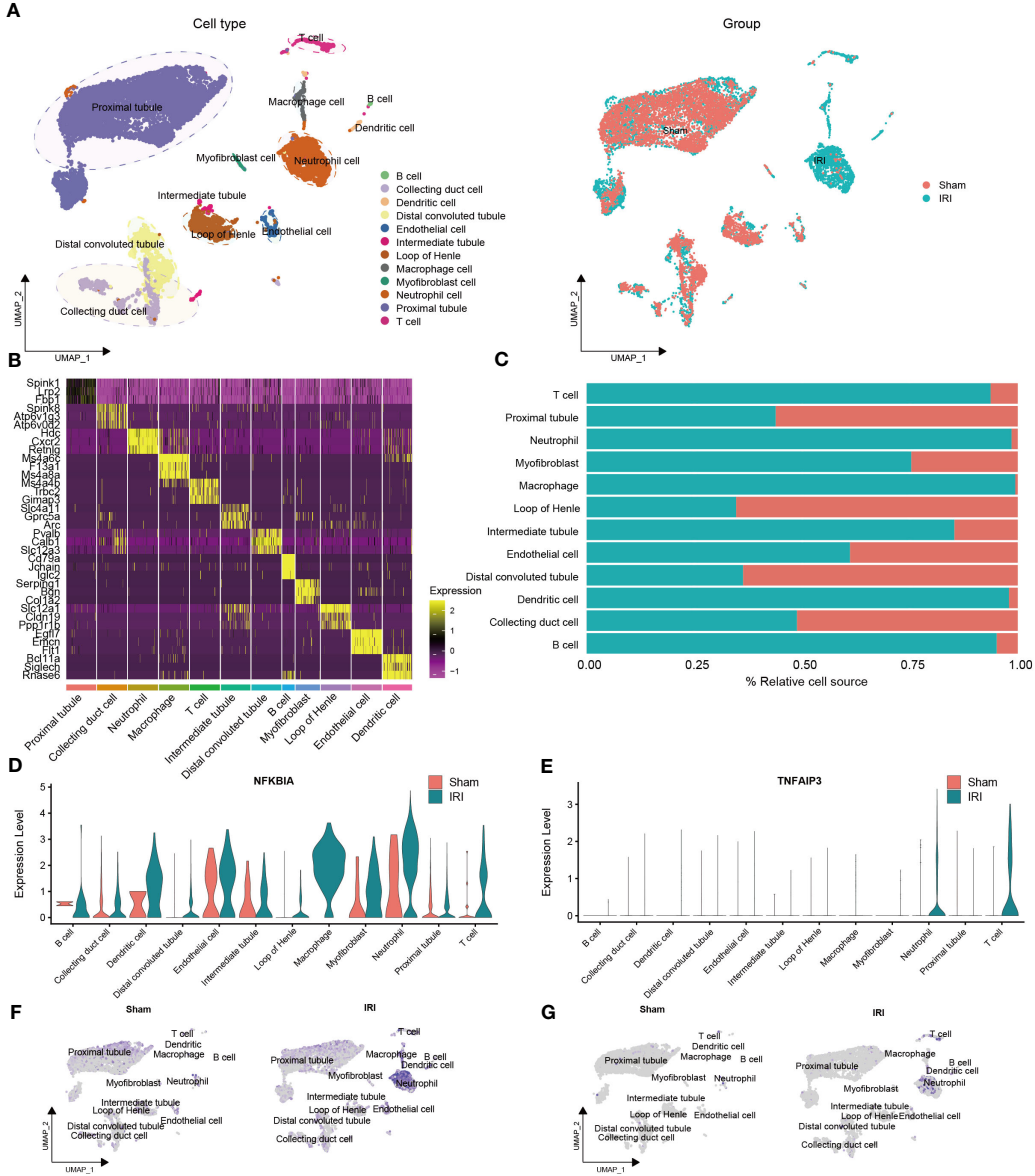
Validation of the NR-DEGs based on scRNA-seq analysis (GSE171639). **(A)** scRNA-seq identified clusters of cells in the sham and IRI kidneys. **(B)** Heatmap showing the top 3 differentially expressed markers in each cluster. **(C)** The proportion of each cell in different groups. **(D-G)** The expression levels of *NFKBIA* **(D, F)** and *TNFAIP3* **(E, G)** in different cells among pre- and post-IRI groups. NR-DEGs, necroptosis-related differentially expression genes; IRI, ischemia-reperfusion injury.

To identify cell types, we combined the CellMarker database and previous studies for mice kidneys (**Figure 4A**). The clusters were annotated as follows: cluster 0, 1, 2, 4, 6, 10, 11, and 13 were annotated as proximal tubule (7,732 cells); cluster 3 as neutrophil cell (1,328 cells); cluster 5 as loop of Henle (874 cells); cluster 7 and 8 as distal convoluted tubule (1,133 cells); cluster 9, 12, 17, and 21 as collecting duct cell (910 cells); cluster 14 as T cell (254 cells); cluster 15 as endothelial cell (239 cells); cluster 16 as macrophage cell (173 cells); cluster 18 as intermediate tubule (102 cells); cluster 19 as myofibroblast cell (81 cells); cluster 20 and 23 as dendritic cell (98 cells); and cluster 22 as B cell (41 cells).

The top three marker genes in each cell subpopulation are presented in **Figure 4B**, and the proportion of different groups in each cell type is shown in **Figure 4C**. It is worth mentioning that T cells, neutrophils, macrophages, dendritic cells, and B cells were predominantly derived from the IRI group, which aligns with the immune infiltration analysis mentioned earlier. We then examined the expression of the five NR-DEGs in each cell type and observed that *NFKBIA, TNFAIP3, MYC*, and *SERTAD1* were mainly expressed in the IRI group (**Figures 4D–G**, **Figures S2C–F**). Specifically, *NFKBIA* exhibited high expression in macrophages, dendritic cells, T cells, and neutrophils; *TNFAIP3* was highly expressed in T cells and neutrophils; *MYC* was predominantly expressed in myofibroblast cells and intermediate tubules; *SERTAD1* was identified as being primarily expressed in myofibroblast cells, macrophages, neutrophils, and endothelial cells. Conversely, *JUN* was predominantly expressed in proximal tubules, intermediate tubules, and endothelial cells (**Figures S2A, B**).

Additionally, the identified cell types of single-cell samples from the GSE193649 dataset were presented in **Figure S3A**. Notably, the expression levels of the five NR-DEGs in GSE193649 dataset were also higher in immune cells of IRI group, such as neutrophils, macrophages, NK cells, and T cells **(Figures S3C–F**, **Figures S4A–F**). These findings suggested that the NR-DEGs are mainly highly expressed in immune cells, which are essential players in the process of IRI.

### 3.6 GSVA and cell-cell communication networks

**Figure 5:**
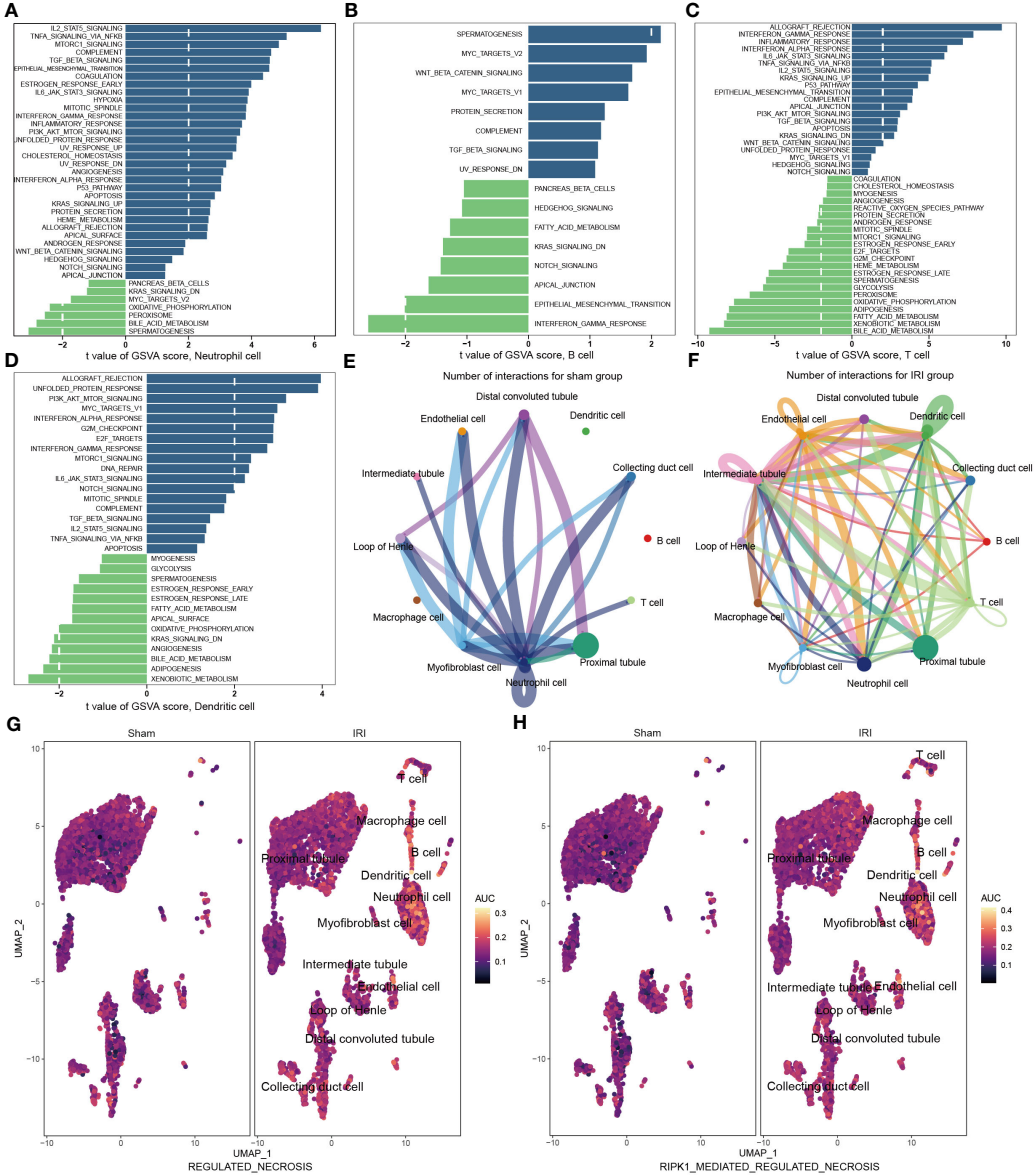
Functional enrichment and cell-cell communication analysis based on scRNA-seq data (GSE171639). **(A–D)** Differences in pathway activities scored in neutrophil cell **(A)**, B cell **(B)**, T cell **(C)**, and dendritic cell **(D)** by GSVA compared with sham and IRI groups. **(E, F)** The number of interactions among different cells in sham **(E)** and IRI groups **(F)**. **(G, H)** UMAP plots showing the necroptotic pathways enrichment scores in sham and IRI groups. GSVA, gene set variation analysis; IRI, ischemia-reperfusion injury.

Following IRI, there was a notable increase in immune cell infiltration and higher relative expression of the NR-DEGs in kidneys. To understand the biological behaviors of these immune subtypes between IRI and sham groups, we performed gene set variation analysis in GSE171639 dataset. The histograms revealed significant enrichment of pathways associated with immune and inflammatory responses, including IL2-STAT5 signaling, TNFA signaling via NF-κB, TGF-β signaling, IL6-JAK-STAT3 signaling, IFN-α/γ response, and PI3K-AKT-mTOR signaling in the IRI group (**Figures 5A–D**, **Figure S2G**). Moreover, the processes of allograft rejection and apoptosis were also more active in the IRI group. Additionally, we compared the enrichment of necroptosis-related pathways using the Molecular Signatures Database (MSigDB, C2, CP: Reactome) with UMAP visualization (**Figures 5G, H**). The regulated necrosis and RIRK1-mediated regulated necrosis pathways were more enriched in immune cells in IRI groups, including neutrophil cells, dendritic cells, macrophage cells, B cells, and T cells. Similar enrichment analysis results of the necroptosis-related pathways were obtained in GSE193649 dataset (**Figures S3G, H**).

To investigate the aggregated cell–cell communication network in the presence or absence of IRI based on the scRNA-seq data, we examined the number and strength of interactions between different cell types. Circle plots (**Figures 5E, F**, **Figures S2H, I**) demonstrated that ligand–receptor interactions were mainly sent from the neutrophil cells, distal convoluted tubules, and myofibroblast cells in the sham group. However, in the IRI group, the communications between the other immune cells, such as B cells, T cells, and dendritic cells, with epithelial and endothelial cells, were significantly increased. Together, these findings further support the notion of a more active necroptosis process and heightened immune responses after IRI, which may play critical roles in the pathogenesis of renal ischemia-reperfusion injury.

### 3.7 Validation of NR-DEGs in IRI model

**Figure 6:**
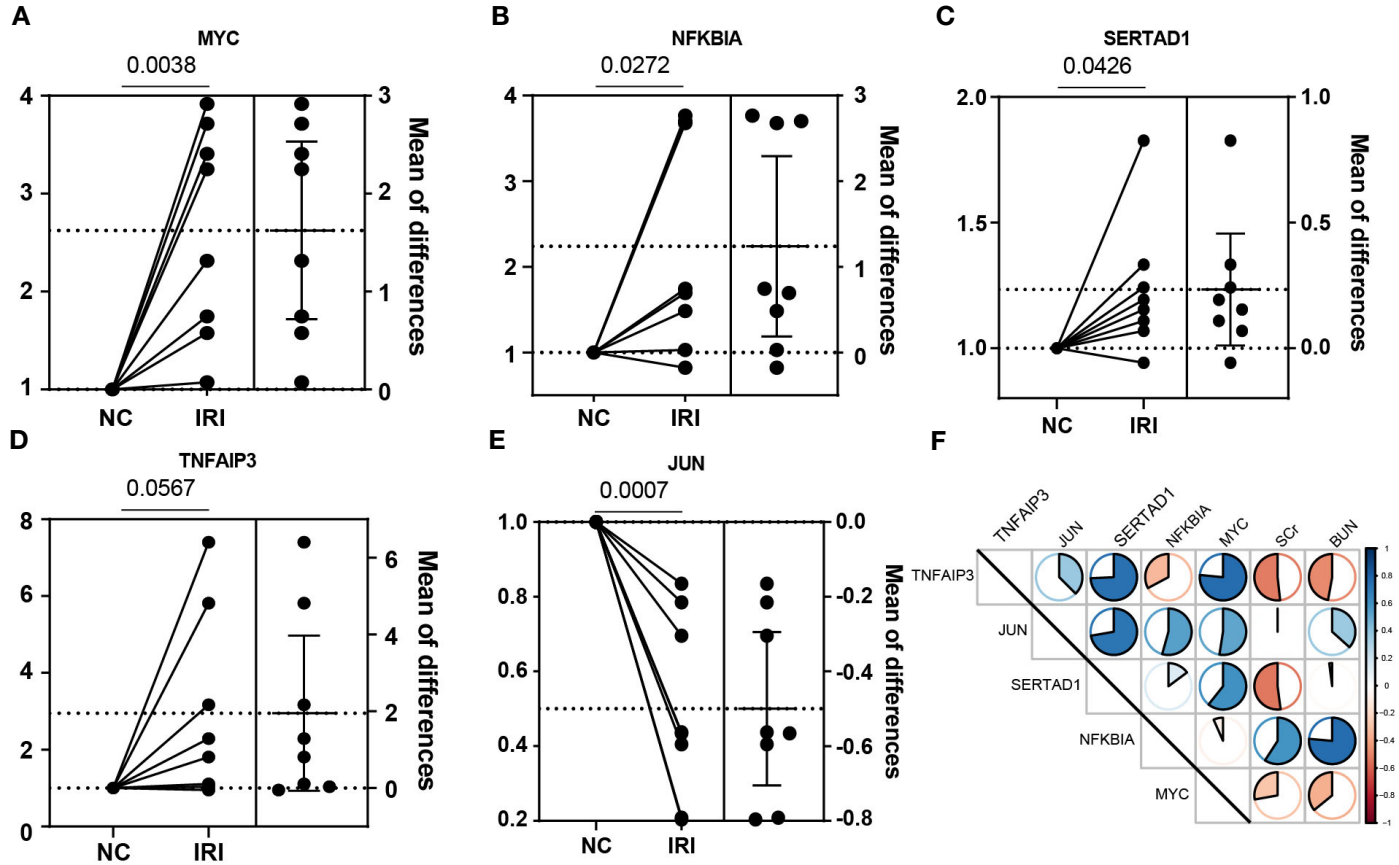
Experimental verification with the mice renal IRI model. **(A–E)** The relative mRNA expression of the NR-DEGs confirmed by qPCR (n = 8). **(F)** The correlation between the expression of NR-DEGs and indexes of renal function (BUN and SCr). IRI, ischemia-reperfusion injury; NR-DEGs, necroptosis-related differentially expression genes; SCr, serum creatinine; BUN, blood urea nitrogen.

To further investigate the important role of the 5 NR-DEGs in the context of kidney IRI, we conducted a model using male C57BL/6N mice. To accurately assess changes in gene expression before and after IR, we removed the right kidney and subjected the left kidney to ischemia-reperfusion treatment. The right kidney served as self-control, minimizing individual differences and potential errors. The PCR results of the same individual’s kidneys, with and without IR, indicated that *MYC*, *NFKBIA*, *SERTAD1*, and *TNFAIP3* were upregulated after IR. However, in contrast to the findings from public databases, *JUN* was significantly downregulated after IR. This discrepancy might be attributed to the differential expression of *JUN* among various cell populations (**Figures 6A–E**). Additionally, we measured the levels of SCr and BUN in mice 24 hours after IR. The results showed consistent correlations with *TNFAIP3* and *NFKBIA*, which had significant contributions to the model established in this study, along with *MYC* (**Figure 6F**).

## 4 Discussion

Despite the improvement of patient and graft survival rates with scientific advances and increasing potency of immunosuppressants, DGF following renal IRI remains an intricate complication after kidney transplantation, resulting in increased morbidity and resource utilization, including longer hospital stays, post-acute care, and higher costs (4, 31). Existing biomarkers like neutrophil gelatinase-associated lipocalin (NGAL) and cystatin C are still not widely applied in clinical settings (32), and proposed models for DGF after deceased-donor transplantation may overestimate its incidence (33). Necroptosis, a caspase-independent form of RN, has been proven to be associated with various models of renal injury, including the oxidative stress-derived IRI (6). Our previous research has demonstrated the role of ferroptosis, another well-studied RN pathway, in acute cell-mediated rejection of KT (34). In the present study, we developed a novel predictive model for DGF occurrence based on necroptosis-related DEGs and revealed the interplay between necroptosis, the hub genes, and immune cells in the process of IRI induced by oxidative stress.

There is growing evidence indicating that necroptosis represents a pivotal component of cell death in renal IRI (35). Markers of necroptosis, including *RIPK1*, *RIPK3*, and *MLKL*, have been shown to be elevated *in vitro* during renal hypoxia/reoxygenation (H/R) injury and *in vivo* during renal IRI studies (36–38). An alleviated renal damage and preserved renal function were detected in the use of small molecule inhibitors like RIPK1 inhibitor Necrostatin-1 (Nec-1) or KO mice (38, 39). To identify potential genetic markers of DGF induced by renal IRI, we intersected DEGs between pre- and post-reperfusion samples with NEGs, resulting in five target NR-DEGs (*NFKBIA*, *TNFAIP3*, *MYC*, *JUN*, *SERTAD1*). All five NR-DEGs were significantly upregulated in post-reperfusion group. Among these, *NFKBIA* (NF-κB inhibitor-α) serves as a direct upstream transcription factor of NF-κB, binding to NF-κB in the cytoplasm and inhibiting its translocation into the nucleus (40, 41). Yatim et al. proposed that NF-κB plays an important role in the activation of RIPK3-induced necroptosis (42). Moreover, it has been demonstrated that *NFKBIA* is upregulated in T- and B-cells during chronic antibody-mediated rejection in KT patients (43). *TNFAIP3* (tumor necrosis factor α-induced protein 3), an ubiquitin-editing enzyme, plays a role in inhibiting the activation of NF-κB and preventing the synthesis of other pro-inflammatory factors, thereby contributing to the regulation of necroptosis (44, 45). Polymorphisms of *TNFAIP3* in humans have been associated with autoimmune diseases and multiple cancers (46). *MYC*, a potent oncogene, is an oncoprotein that regulates various cellular processes (47). It functions as an antinecroptotic regulator by inhibiting the formation of RIPK3-RIPK1 complex (48). In addition, *JUN* is an important component of activating protein-1 (AP-1), which has been proven to regulate cell death and survival (49). C-Jun, the most intensively studied member of the JUN family, is associated with the necroptotic pathway, specifically the RIPK3-JNK-BNIP3 (c-Jun N-terminal kinase-BCL2 Interacting Protein 3) axis (50). *SERTAD1*, also known as SERTA domain-containing protein 1, belongs to the Sertad family (51) and functions as an oncoprotein that significantly contributes to oncogenesis and programmed cell death (PCD), including necroptosis (52). Additionally, *SERTAD1* has been implicated in promoting cell survival in response to the induction of reactive oxygen species by facilitating the ubiquitination of apoptosis signal-regulating kinase1 (ASK1) (53).

To elucidate distinct patterns of necroptosis modification, we conducted the unsupervised consistent clustering analysis based on the NR-DEGs. The renal samples were categorized into C1 and C2 clusters, where all five NR-DEGs exhibited higher expression in the C1 cluster. Notably, the C1 cluster showed a lower incidence of DGF and a higher proportion of recipients receiving kidneys from BD donors. These findings suggest that although the NR-DEGs are upregulated after IRI, they may confer a protective effect against renal damage and contribute to reduced complications, as we mentioned above.

The analysis of functional enrichment revealed that the IRI-related DEGs exhibited significant enrichment in the regulation of RNA polymerase II transcription and MAPK signaling, IL-17 signaling, and TNF signaling pathways. Similarly, the GSVA analysis between the two necroptosis-related clusters suggested a strong association of the C1 group with multiple immune-related pathways, including MAPK signaling, T cell receptor signaling, Toll-like receptor (TLR) signaling pathways, and apoptosis pathway. TNFα is a well-studied trigger of necroptosis cell death (6). Upon TNF binding to its receptor, TNF receptor 1 (TNFR1), various downstream molecules are recruited to form complex I, providing a platform to determine cell survival, apoptosis, or necroptosis (54). *In vitro* studies have demonstrated that a combination of TNFα, interferon (IFN)-γ, and the weak inducer of TNF signaling (TWEAK) can induce necroptosis in TECs (55). TLRs are constitutively expressed in various renal cells. Activation of TLRs induces the interaction between downstream molecules and complex IIb (composed of RIPK1 and RIPK3), leading to MLKL-dependent necroptosis (56). These findings suggest that the proinflammatory pathways associated with necroptosis may be significantly inhibited in C1 cluster.

Since IRI is inevitable in surgical procedures of KT and is the primary cause of DGF, it is of utmost clinical importance to identify patients at higher risk of DGF early and accurately. In the present study, we developed an early predictive model based on the five NR-DEGs through LASSO regression analysis. The performance of this predictive model was robust and satisfactory in both the discovery cohort (AUROC = 0.798) and the whole cohort (AUROC = 0.749). To explore the underlying mechanism, we stratified the renal samples into low- and high-risk groups and examined the immune cell infiltration. High-risk samples exhibited higher infiltration of memory B cells, naïve CD4 T cells, and gamma delta T cells, which were also prevalent in samples of C2 cluster characterized by a higher incidence of DGF. This suggests that specific immune cells potentially served as a bridging link function between necroptosis and the progression of DGF. Therefore, we conducted single-cell data analysis to further corroborate the validity of our model genes.

To substantiate our findings, we utilized two published single-cell datasets of mouse renal samples obtained with or without IR. In the IRI group, immune cells such as T cells, B cells, dendritic cells, and neutrophils were more abundant, concomitant with higher expression levels of the NR-DEGs, particularly in neutrophils and macrophages. During renal IRI, damaged cells release damage-associated molecular patterns and proinflammatory cytokines such as TNFα and IL-1α, which can bind to cell surface receptors like TLRs, resulting in dendritic cell migration and activation of T cells and macrophages as observed in our intercellular communication (6, 57). Previous research has proved that B-cell and T-cell deficient mice are protected from renal IRI (58). While, necroptosis, as an immunity-related programmed cell death, regulates the proliferation of lymphocytes and is crucial for their survival (59). RIP1 is essential for B cell development and is more highly expressed in immature B cells and peripheral mature B cells (60). Zhang et al. indicated that the proliferation response of RIP^-/-^ B cells induced by TLR and lipopolysaccharide (LPS) was reduced compared to RIP^+/+^ B cells (61). Besides, caspase-8 is the key molecule of T cell homeostasis, and the impaired T cell proliferation in caspase 8-deficient mice can be rescued by blocking RIP1 with Nec-1 or through gene knockout, indicating that the necroptotic signaling in T cells is regulated by caspase-8 (59, 62, 63). Recent studies demonstrated that the RIRK1-dependent necroptosis is reduced during macrophage cell differentiation (64), while TNFα derived from macrophages induces necroptosis (65). Additionally, it has been suggested that IR-induced AKI depends on the migration of neutrophils into kidneys (66). Necroptosis in neutrophils can be induced by activating TLRs, IFN-α receptors, TNF receptors, and other factors (67, 68). Early research reported that inhibiting XIAP (X-linked inhibitor of apoptosis family of protein) resulted in increased ubiquitylation of neutrophil RIPK1 (69), restricted RIPK3-dependent cell death in dendritic cells (70), and limited macrophages necroptosis (71).

We further analyzed the functional differences between immune cells before and after IR. It is worth noting that the necroptosis-related pathways, including TNFα signaling and IFN response, were enriched in these immune cells after IR (6, 54), supporting the biological plausibility of our findings. *MYC*, one of the NR-DEGs, known as a negative regulator of necroptosis (47), showed increased activity in the IRI group in our study, possibly due to the proteasomal degradation of *MYC* stimulated by RIPK3. In addition, previous studies have also linked necroptosis to an immune response in various diseases, encompassing IL6-JAK-STAT3 and IL2-STAT5 signaling pathways (72, 73). Activation of TAK1 (TGF-β activated kinase 1) can induce RIPK3-dependent necroptosis (74). RIPK1 facilitates the reciprocal stimulations between TAK1 and RIPK3, in turn mediates TAK1-RIPK1-RIPK3 binding, which decides whether the necroptosis occurs or not (75). Meanwhile, the enrichment of “regulated necrosis” and “RIPK1 mediated regulated necrosis” was observed in the IRI group, especially in proinflammatory cells. Together, these findings are consistent with prior studies and highlight the important contributions of immune cells in the regulation of necroptosis after renal IR. The results not only support the validity of the prediction models but also provide potential targets for addressing necroptosis-related renal oxidative stress damage.

Our findings were experimentally validated using the mouse renal IRI model. We observed that all the identified NR-DEGs were upregulated after IR, except for *JUN*. This confirmed the role of necroptotic progress. Furthermore, the negative correlations between *TNFAIP3* and *MYC* with kidney function markers also indicated a potential protective action of these genes in necroptosis (45, 48). While, the *NFKBIA* was associated with higher BUN and SCr, likely due to its role in suppressing cell survival induced by NF-kB (76).

The primary strengths of this study lie in the construction of a predictive model for DGF based on the concept of necroptosis. The study successfully demonstrated the correlation between NR-DEGs, oxidative stress, and immune cells, providing valuable insights into the role of necroptotic cell death in renal IRI. Nevertheless, a portion of dead cells were filtered out to improve the accuracy of cell identification among scRNA-seq analysis, which may lead to the loss of information related to necroptosis. Additionally, to ensure the reliability and applicability of the prediction model, further validation using data from multicenter and KT patients is necessary. Furthermore, considering the potential of NR-DEGs as targets for preventing DGF, it is imperative to conduct an experimental investigation to understand the cellular and molecular mechanism through which these genes influence renal IRI. Such investigations could pave the way for potential therapeutic interventions to improve outcomes in kidney transplantation.

## 5 Conclusions

To summarize, our study has identified five NR-DEGs and confirmed their expression levels by utilizing scRNA-seq data and a mouse IRI model. We developed a novel diagnostic model based on the five genes, enabling us to predict the occurrence of DGF more accurately. Furthermore, we observed distinct differences in necroptotic immune cell profiles and proinflammatory responses between the low- and high-risk groups, highlighting the clinical relevance of our findings. By offering insights into the underlying mechanisms and potential predictive markers, our study may aid clinicians in decision-making and provide potential therapeutic strategies for the prevention of IRI and DGF in kidney transplantation.

## Data availability statement

Publicly available datasets were analyzed in this study. This data can be found here: GSE43974, GSE171639, GSE126805, and GSE193649 (https://www.ncbi.nlm.nih.gov/geo/).

## Ethics statement

The animal study was approved by The Ethics Committee of Beijing Chaoyang Hospital Affiliated to Capital Medical University. The study was conducted in accordance with the local legislation and institutional requirements.

## Author contributions

SL: Conceptualization, Data curation, Methodology, Visualization, Writing – original draft. WZ: Conceptualization, Data curation, Methodology, Visualization, Writing – review & editing. XH: Conceptualization, Supervision, Writing – review & editing.
