# Supplementary material for: Comprehensive analysis of necroptosis-related genes in renal ischemia-reperfusion injury
Source: Front Immunol. 2023 Oct 27;14:1279603. doi: 10.3389/fimmu.2023.1279603 (PMC10641517; doi:10.3389/fimmu.2023.1279603)
Supplement: Supplementary file 1 [file Image_1.pdf]

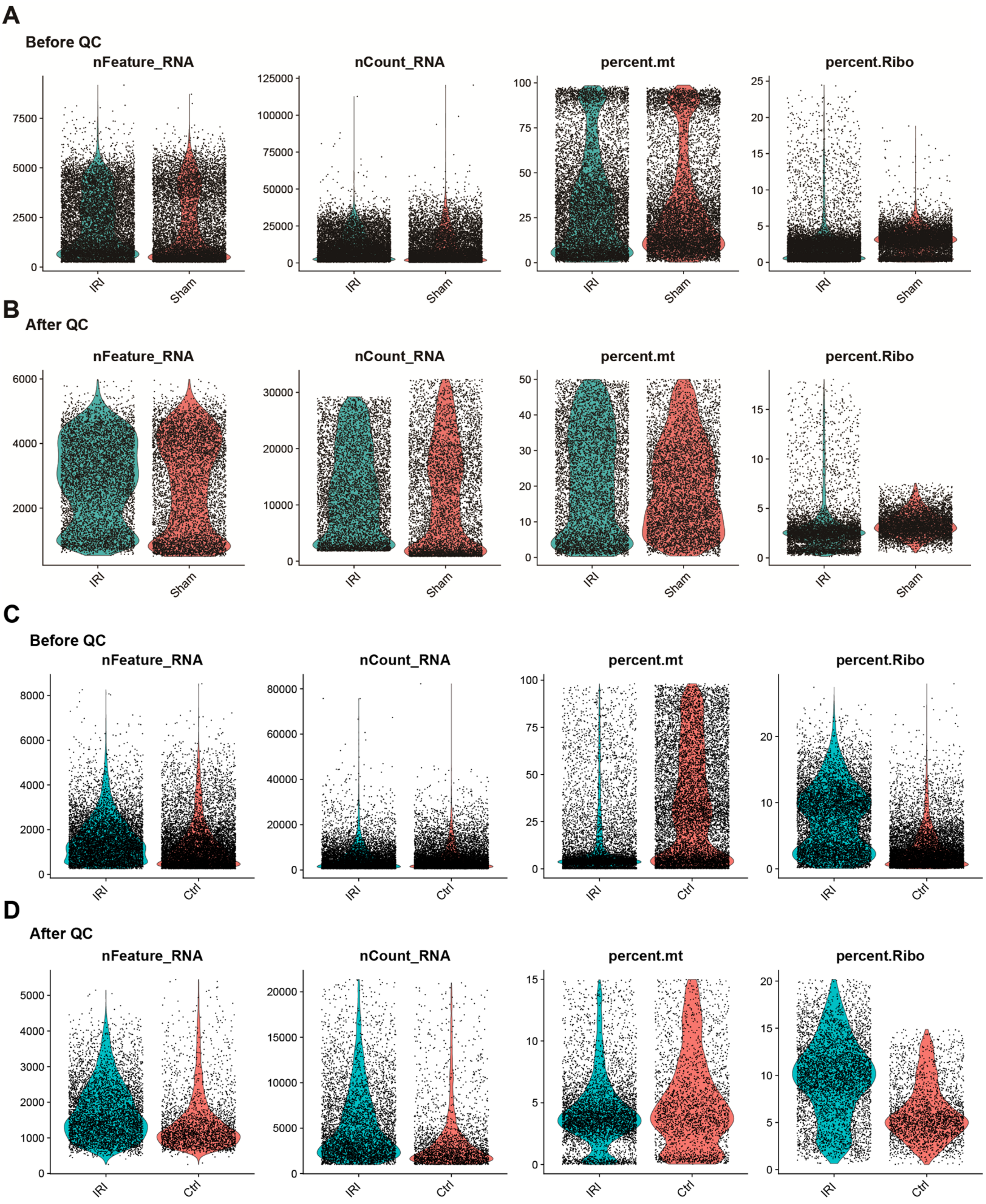


Fig. S1. Quality control (QC) of sing-cell RNA sequencing data. (A, B) Violin plots of the total number of genes detected (nFeature_RNA), number of transcripts (nCount_RNA), and percentage of mitochondrial genes (percent.mt) and ribosomal genes (percent.Ribo) for each cell before (A) and after (B) QC of samples from the GSE171639 dataset. QC was performed by removing low-quality cells and doublets by the cell subset at nFeature_RNA < 6,000 & percent.mt < 50; (C, D) Violin plots of the nFeature_RNA, nCount_RNA, percent.mt, and percent.Ribo for each cell before (C) and after (D) QC of samples from the GSE193649 dataset. QC was performed by the cell subset at nFeature_RNA < 8,000 & percent.mt < 15.
