# Supplementary material for: Comprehensive analysis of necroptosis-related genes in renal ischemia-reperfusion injury
Source: Front Immunol. 2023 Oct 27;14:1279603. doi: 10.3389/fimmu.2023.1279603 (PMC10641517; doi:10.3389/fimmu.2023.1279603)
Supplement: Supplementary file 2 [file Image_2.pdf]

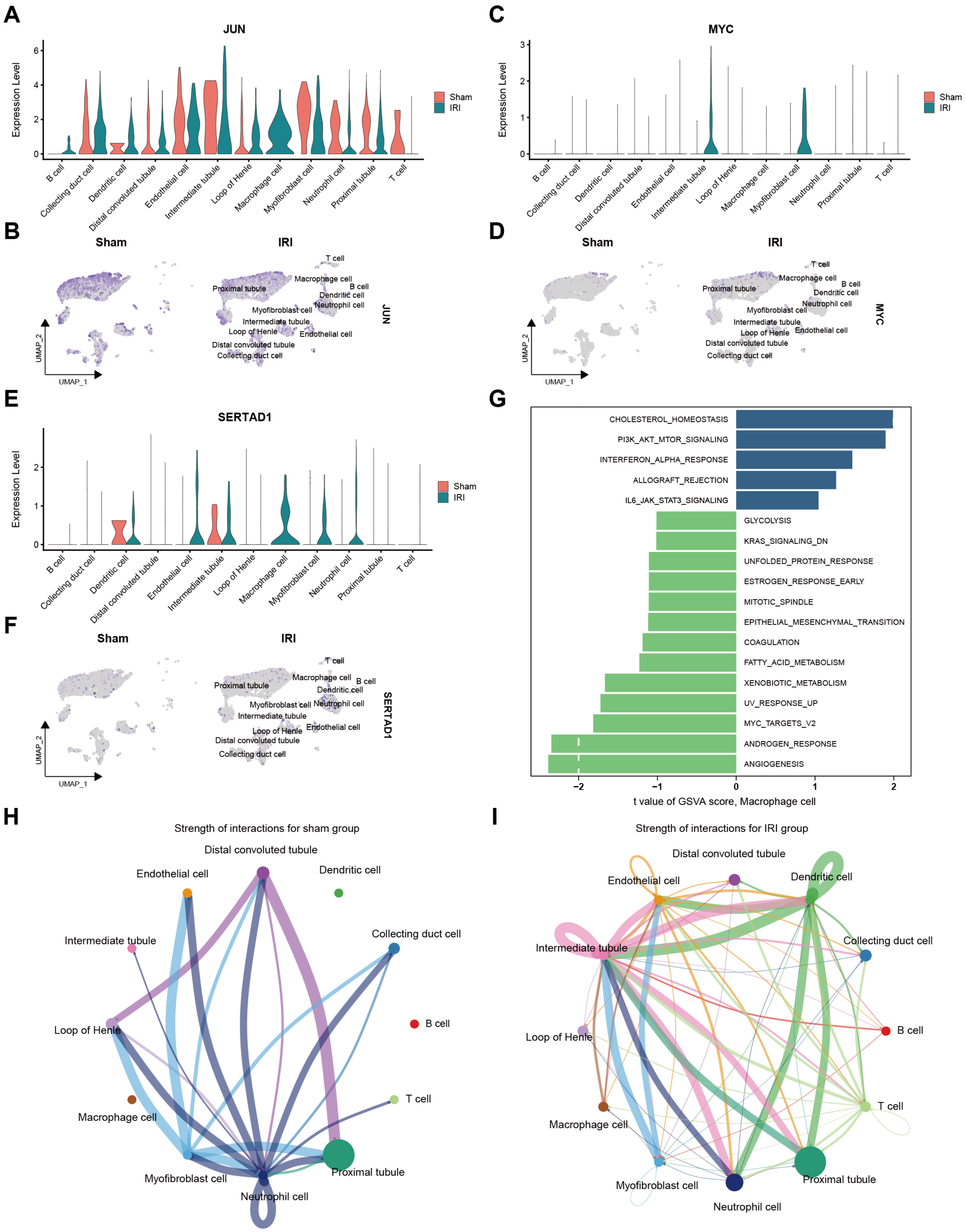

Fig. S2. Validation of the NR-DEGs based on scRNA-seq analysis (GSE171639). (A-F) The expression levels of *JUN* (A, B), *MYC* (C, D), and *SERTAD1* (E, F) in different cells among pre- and post-IR groups. (G) Differences in pathway activities scored in macrophage cells by GSVA compared with sham and IRI groups. (H, I) The strength of interactions among different cells in sham (H) and IRI groups (I). NR-DEGs: necroptosis-related differentially expression genes; IRI: ischemia-reperfusion injury; GSVA: gene set variation analysis.
