# Supplementary material for: Comprehensive analysis of necroptosis-related genes in renal ischemia-reperfusion injury
Source: Front Immunol. 2023 Oct 27;14:1279603. doi: 10.3389/fimmu.2023.1279603 (PMC10641517; doi:10.3389/fimmu.2023.1279603)
Supplement: Supplementary file 3 [file Image_3.pdf]

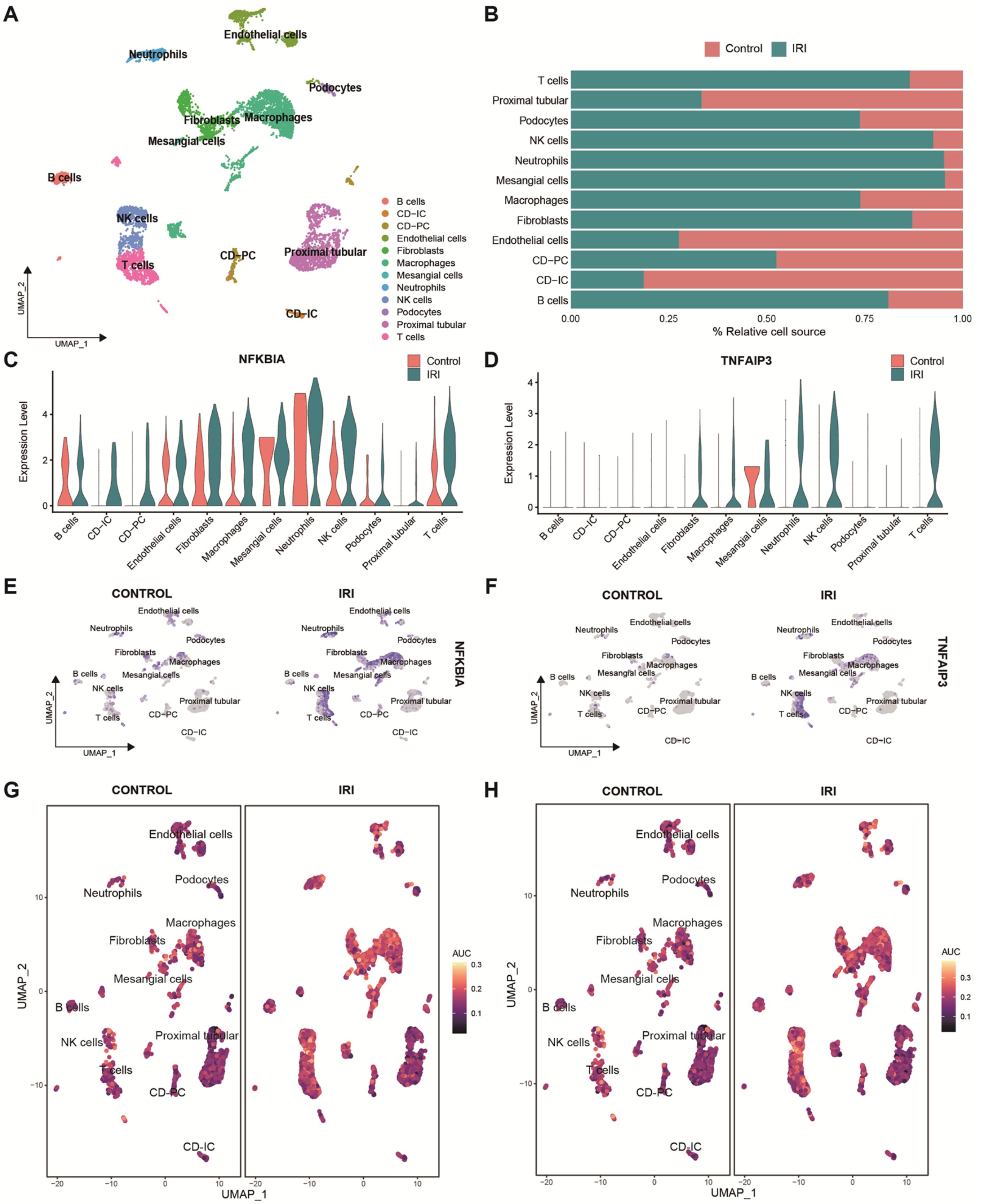

Fig. S3. Validation of the NR-DEGs based on scRNA-seq analysis (GSE193649). (A) scRNA-seq identified clusters of cells in the control and IRI groups. (B) The proportion of each cell in different groups. (C-F) The expression levels of *NFKBIA* (C, E) and *TNFAIP3* (D, F) in different cells among control and IRI groups. (G, H) UMAP plots showing the enrichment scores of regulated necrosis (G) and RIPK1-mediated regulated necrosis (H) pathways in control and IRI groups. CD-IC: collecting duct intercalated cells; CD-PC: collecting duct principal cells; IRI: ischemic-reperfusion injury.
