# Supplementary material for: Comprehensive analysis of necroptosis-related genes in renal ischemia-reperfusion injury
Source: Front Immunol. 2023 Oct 27;14:1279603. doi: 10.3389/fimmu.2023.1279603 (PMC10641517; doi:10.3389/fimmu.2023.1279603)
Supplement: Supplementary file 4 [file Image_4.pdf]

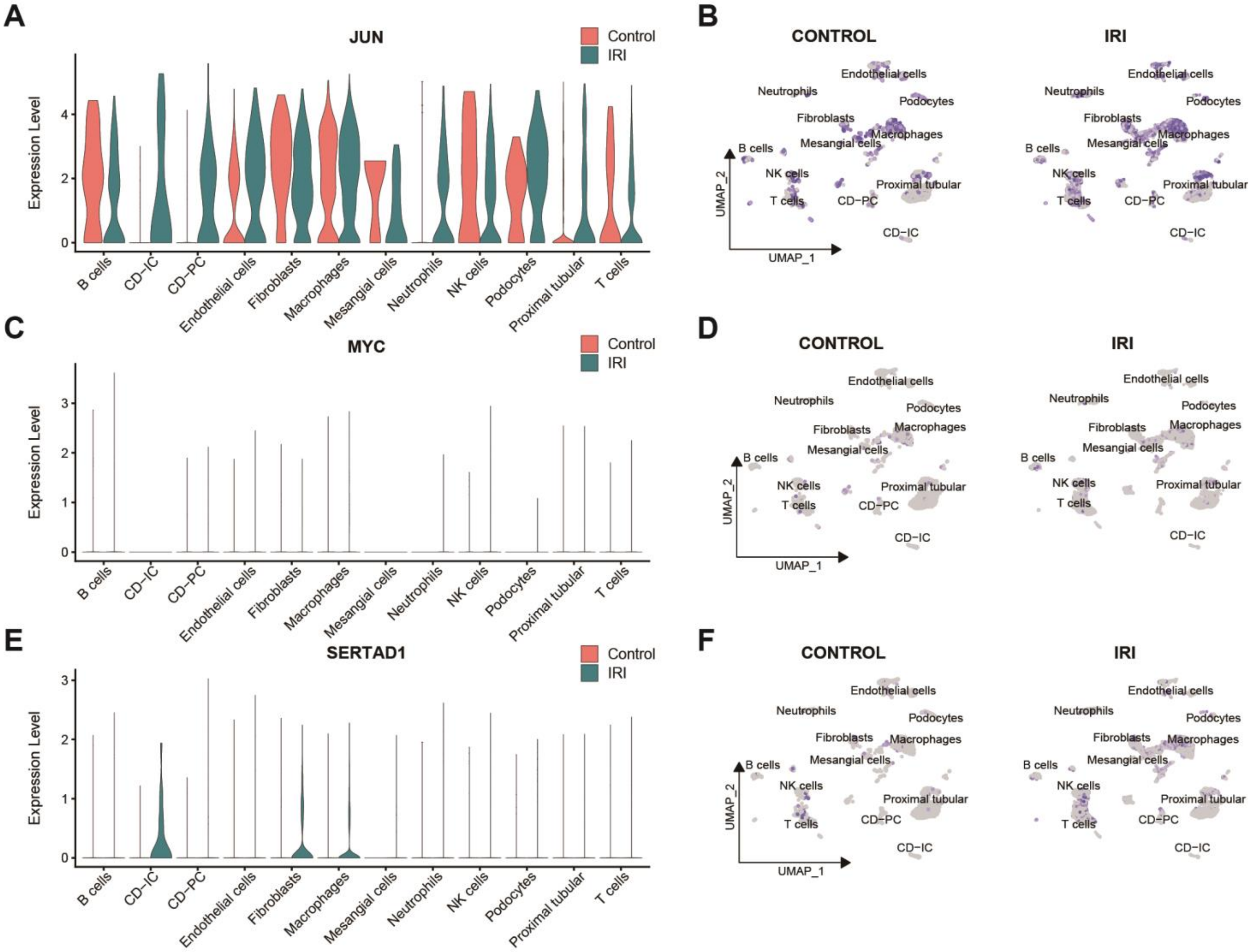

Fig. S4. The expression levels of *JUN* (A, B), *MYC* (C, D), and *SERTAD1* (E, F) in different cells among control and IRI groups in the GSE193649 dataset. IRI: ischemic-reperfusion injury; CD-IC: collecting duct intercalated cells; CD-PC: collecting duct principal cells.
